# Ultrastructural Study of an Atypical Case of Infertility

**DOI:** 10.1100/tsw.2007.151

**Published:** 2007-04-09

**Authors:** Ernesto Sánchez Sánchez, Carmen Tejedor Mardomingo

**Affiliations:** Department of Urology, University Hospital “Virgen Macarena”, Seville, Spain

**Keywords:** semen, sperm tail, male, spermatozoa, stump tail, short tail

## Abstract

An atypical case of infertility associated with severe sperm abnormalities is presented. A 29-year-old man with 4 years of primary infertility had no history of significant illness, and no hereditary pathology or male infertility existed in his family. Physical examination of the patient showed no pathological findings. The analyses of four semen samples showed: sperm count, 67-83 10/ml; 0% motility. The morphological analysis showed absence of flagellum, 14-16%; short-tail spermatozoa, 45-64%; coiled tails, 12-17%; and an abnormal proportion of spermatids and spermatocytes. Normal spermatozoa were found in 11-16%. Endocrine profile was found within the normal range. Testicular biopsy revealed impaired spermatogenesis. Scanning electron microscopy revealed sperm heads with intact nuclei and acrosomal regions. To our surprise, some of the stunted tails were uniflagellate. To our knowledge, this is a very uncommon case of sperm tail defect.

## INTRODUCTION

Many cases of male infertility are associated with severe sperm tail abnormalities. The first reports of a syndrome characterized by stunted tails and sperm immobility was reported in rabbits[1] and bulls[2]. Electron microscopy demonstrated that the tail defect results in a blocked formation of the flagellum, generating the absence of axonema and accessory fibers[3]. This syndrome was known as "stump defect". Baccetti et al. described a tail defect in humans[4], in which sperm immobility was also due to stunted tails, but this case was mainly characterized by the presence of biflagellate stunted tails. This abnormality was then called "short tail" defect. Subsequently, Baccetti et al.[5] reported eight human sperm cases of stunted tails, in which half were classified as stump defect and the other half as short tail defect. This classification was fundamentally based on two characteristics: the presence of an extremely low sperm number ($11–19 \times 10^6$ ml$^{-1}$) and biflagellate tails in "short tail" defect, and low sperm ($4–52 \times 10^6$ ml$^{-1}$) and one stunted tail in "stump tail" defect. To our knowledge, only two other new cases of stunted sperm tails have been further reported[6,7]. We report a new case of this extraordinary unusual defect in human sperm tail. This case will help to characterize these syndromes in a better way.

## CASE REPORT

A 29-year-old man presented to our reproduction department in order to be investigated for a 4-year primary infertility. He had no history of significant illness and neither the patient, nor anyone else in his family, had cilia respiratory diseases. His parents are not consanguineous and he has six bothers and three sisters with children. Physical examination of the patient showed no pathological findings.

### Serum and Sample Analysis

Two serum samples were analyzed for follicle stimulating hormone (FSH), luteinizing hormone (LH), and testosterone (T), and were assayed by routine radioimmunoassay.

### Semen Analysis

Four semen samples were collected by masturbation after 3 days of sexual abstinence and were analyzed within 1 h of collection. PH, sperm counts, motility, live forms, and morphology were appraised with standardized methods (World Health Organization recommended procedure, 1999). Sperm morphology was assessed after Papanicolau staining on 100 spermatozoa according to the classification of the World Health Organization.

### Testes Analysis

A biopsy was performed on both testes, and samples were fixed in Bouin´s solution, embedded in paraffin, and sections stained with hematoxylin-eosin.

### Electron Microscopy Procedure

The semen sample was diluted (1:3) with Ham´s F10 medium and spermatozoa were separated by centrifugation. The pellets were fixed for 2–4 h with 3% glutaraldehyde and postfixed for 2 h in 1.3% osmium tetroxide. The spermatozoa were fixed in suspension with buffer washes between and after fixation steps. Sperm cells were subsequently sedimented on poly-L-lysine–coated glass slide fragments and were dehydrated in a graded series of ethanol followed by absolute acetone, dried in Balzers CDP 030 (Balzers Union Ltd, Balzers, Liechtenstein) critical point dying apparatus using $CO_2$ as transmission fluid, coated with gold-palladium in a Balzers Union CDP 040, and observed in a scanning electron microscope.

## RESULTS

The analyses of four semen samples showed the following results: sperm count, 67-83 $10^6$/ml (>20 × $10^6$/ml); 35% (>50%) alive forms; and 0% (>50%) motility. No abnormality was found in biochemical parameters in seminal plasma. The morphological analysis revealed mainly tail disturbances: absence of flagellum, 14–16%; short-tail spermatozoa, 45–64% (Fig 1); coiled tails, 12–17%; and an abnormal proportion of spermatids and spermatocytes. Normal spermatozoa were found in 11–16% (>30%).

Endocrine profile was found within the normal range: FSH, 6.4 (2–8 mUI/ml); LH, 7.2 (2–12 mUI/ml); T, 28 (10–35 nmol/l).

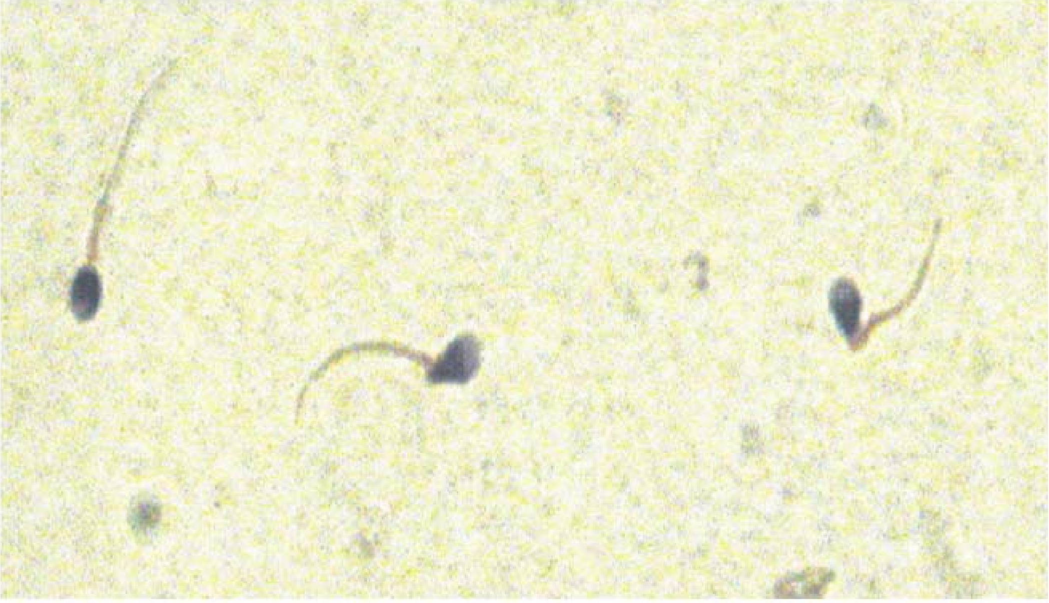

**FIGURE 1.** Short-tail spermatozoa.

Testicular biopsy revealed impaired spermatogenesis. We also found reduced tail-length spermatozoa in the seminiferous tubules lumen with a high percentage of degenerative spermatocytes and spermatids.

Scanning electron microscopy confirmed the abnormalities observed with optic microscopy (Fig. 2), and revealed sperm heads with intact nuclei and acrosomal regions. Surprisingly, some spermatozoa had uniflagellate tails.

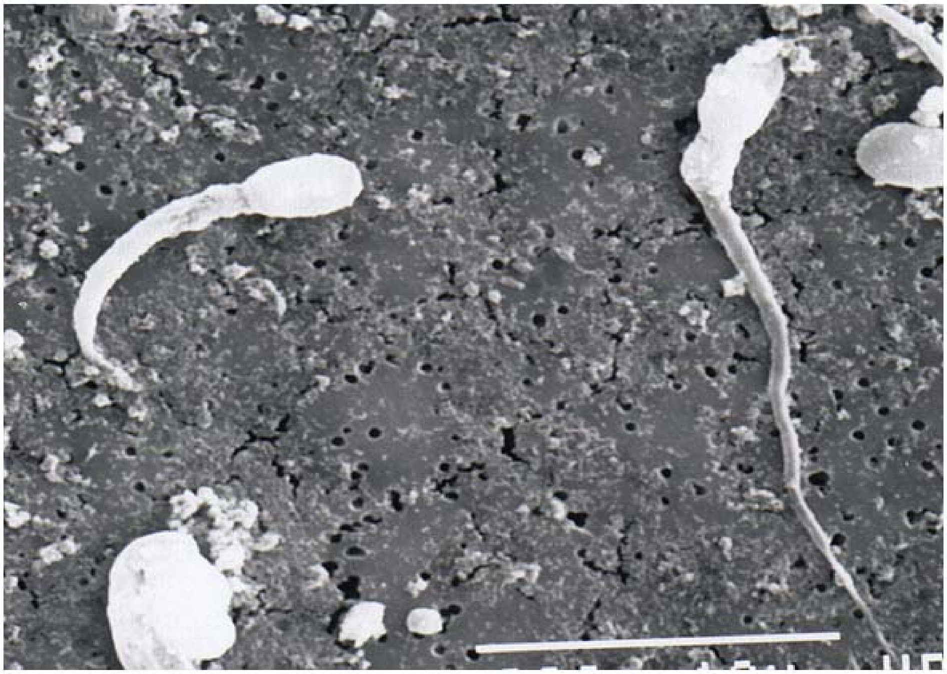

**FIGURE 2.** The photography shows a short-tail spermatozoa on the left, and a normal-length spermatozoa tail on the right.

## DISCUSSION

The semen analyzed showed some, but not all, of the morphological defects described by Baccetti et al.[4,5] for the stump tail syndrome. The spermatozoa showed a complete immobility and most of them had a reduced tail. However, it was found that the number of spermatozoa was not reduced and that the total sperm population was not affected by the extremely reduced length of the tails. According to the criteria of Baccetti et al.[4,5], the syndrome can be described as stump tail syndrome.

Our case confirms the testicular dysfunction of spermiogenesis as the origin of these kinds of tail abnormalities. However, the true origin of this defect remains unknown. Sperm and cilia disturbances are often reported to be associated and genetically determined; in fact, familial cases have been described in brothers[8]. Conversely, our patient had no familial history; neither other cilia disturbances nor mail infertility. Therefore, this observation would suggest the possibility of an acquired defect or mosaics of genes that determine cilia and flagella axonemes.
